# First Mitochondrial Genome from Nemouridae (Plecoptera) Reveals Novel Features of the Elongated Control Region and Phylogenetic Implications

**DOI:** 10.3390/ijms18050996

**Published:** 2017-05-05

**Authors:** Zhi-Teng Chen, Yu-Zhou Du

**Affiliations:** 1 School of Horticulture and Plant Protection & Institute of Applied Entomology, Yangzhou University, Yangzhou 225009, China; wstcczt@gmail.com; 2 Joint International Research Laboratory of Agriculture and Agri-Product Safety, The Ministry of Education, Yangzhou University, Yangzhou 25009, China

**Keywords:** Plecoptera, stoneflies, mitochondrial genome, control region, phylogeny

## Abstract

The complete mitochondrial genome (mitogenome) of *Nemoura nankinensis* (Plecoptera: Nemouridae) was sequenced as the first reported mitogenome from the family Nemouridae. The *N. nankinensis* mitogenome was the longest (16,602 bp) among reported plecopteran mitogenomes, and it contains 37 genes including 13 protein-coding genes (PCGs), 22 transfer RNA (tRNA) genes and two ribosomal RNA (rRNA) genes. Most PCGs used standard ATN as start codons, and TAN as termination codons. All tRNA genes of *N. nankinensis* could fold into the cloverleaf secondary structures except for *trnSer* (*AGN*), whose dihydrouridine (DHU) arm was reduced to a small loop. There was also a large non-coding region (control region, CR) in the *N. nankinensis* mitogenome. The 1751 bp CR was the longest and had the highest A+T content (81.8%) among stoneflies. A large tandem repeat region, five potential stem-loop (SL) structures, four tRNA-like structures and four conserved sequence blocks (CSBs) were detected in the elongated CR. The presence of these tRNA-like structures in the CR has never been reported in other plecopteran mitogenomes. These novel features of the elongated CR in *N. nankinensis* may have functions associated with the process of replication and transcription. Finally, phylogenetic reconstruction suggested that Nemouridae was the sister-group of Capniidae.

## 1. Introduction

Nowadays, mitochondrial genome (mitogenome) has been one of the most popular molecules widely used in insect taxonomy, population genetics, evolutionary biology and phylogenetics [1]. Generally, an insect mitogenome is a double strand circular molecule, ranging from 14–20 kb in length. It usually contains a typical set of 37 genes: 13 protein-coding genes (PCGs), 22 transfer RNA (tRNA) genes and two ribosomal RNA (rRNA) genes [2,3]. There is also a non-coding control region (CR) in mitogenomes, which is involved in the initiation and regulation of transcription and replication of the mitogenome [4,5,6]. The CR is the most variable region concerning A+T content and length, and some structural elements are expected to be present in a CR: (1) a poly-T stretch near the 5′ end of the CR; (2) a poly-[TA(A)]*_n_* stretch following the poly-T stretch; (3) conserved stem-loop (SL) structures with a 5′ flanking TATA and a 3’ flanking G(A)*_n_*T motif; and (4) a G+A-rich region located downstream of the CR [7]. Functional information on replication derived from these structures has been well discussed, but the transcription features of insect mitogenomes are still little known [6,7,8,9].

The Plecoptera (stoneflies) are a group of ancient insects, which are vital in the reconstruction of the evolutionary history of insects, and they are important bioindicators of water quality [10]. To date, 15 complete or near complete plecopteran mitogenomes have been reported, and relevant attempts have been made to rebuild the phylogeny of Plecoptera based on the increasing mitogenomic data [11,12,13,14,15,16,17,18,19,20,21,22,23,24]. However, the phylogenetic position of Plecoptera in Insecta and the phylogenetic relationship among stoneflies are still controversial.

To facilitate the study of mitogenome phylogeny in Plecoptera, we report the complete mitogenome of *Nemoura nankinensis*, which was the first sequenced mitogenome from Nemouridae. In this study, the organization, nucleotide composition, codon usage, secondary tRNA structures, and novel features of the elongated CR in the *N. nankinensis* mitogenome were analyzed. Finally, the phylogenetic relationships of *N. nankinensis* and other stoneflies were reconstructed based on PCG sequences.

## 2. Results and Discussion

### 2.1. Genome Annotation and Base Composition

**Figure 1:**
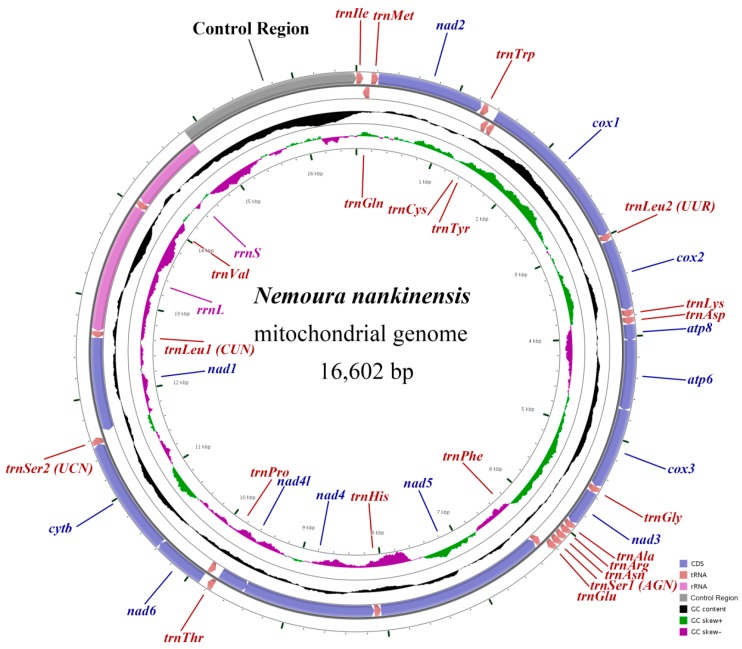
Mitochondrial map of *Nemoura nankinensis*. Genes outside the map are transcribed in a clockwise direction, whereas those inside the map are transcribed counterclockwise. The second circle shows the GC content and the third shows the GC skew. GC content and GC skew are plotted as the deviation from the average value of the entire sequence.

The complete mitogenome of *N. nankinensis* was 16,602 bp in length, which was larger than any other reported stonefly mitogenomes. It contained the 37 typical mitochondrial genes (13 PCGs, 22 tRNAs and two rRNAs) and a large noncoding control region; 23 genes (nine PCGs and 14 tRNAs) were located on the majority strand (J-strand) and 14 genes (four PCGs, eight tRNAs, and two rRNAs) were on the minority strand (N-strand) (Figure 1, Table 1). The highly-conserved gene arrangement of the *N. nankinensis* mitogenome was identical with other stoneflies as well as the model insect, *Drosophila yakuba*, which had the putative ancestral arthropod mitogenome [25]. The *N. nankinensis* mitogenome contained 36 overlapping nucleotides that were 1–8 bp in length and located in 11 pairs of neighboring genes. The longest overlap (8 bp) was located between *trnCys* and *trnTrp*. Except for the large control region, a total of 72 intergenic nucleotides (IGN) were found in 13 locations, ranging in size from 1 to 31 bp.

In the *N. nankinensis* mitogenome, the A+T content of the whole mitogenome, PCGs, tRNAs, rRNAs and the control region was 71.2%, 69.0%, 71.4%, 73.5% and 81.9%, respectively (Table 2). For the 37 genes, the A+T content ranged from 60.6% in *trnTyr* to 90.9% in *trnGlu*, showing a bias for the A and T nucleotides. The AT skew and GC skew of the *N. nankinensis* mitogenome were calculated and showed a biased use of A and C nucleotides (Table 2). For the J-strand, the AT skew of PCGs was negative and the GC skew of tRNA genes was positive, which was inconsistent with the strand bias of most other insects (positive AT skew and negative GC skew for the J-strand) [26].

### 2.2. Protein-Coding Genes, Transfer RNA and Ribosomal RNA Genes

**Figure 2:**
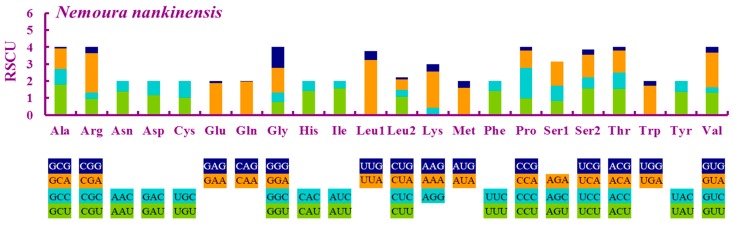
Relative synonymous codon usage (RSCU) in the *Nemoura nankinensis* mitogenome. Codon families are indicated below the *X*-axis.

The 13 PCGs of *N. nankinensis* were similar in length and arrangement to other sequenced stonefly mitogenomes. Eleven PCGs initiated with the standard start codon ATN (ATT and ATG), while *cox1* used CCG, and *nad1* used TTG as a start codon. Ten PCGs had complete termination codons (TAA or TAG), whereas *cox1*, *cox2* and *nad5* ended with the incomplete termination codon T, which could be completed by post-transcriptional polyadenylation [27]. The relative synonymous codon usage (RSCU) values of the *N. nankinensis* mitogenome were calculated and illustrated, indicating the five most frequently used codons: TTA (Leu), CGA (Arg), GTA (Val), AAA (Lys) and CAA (Gln) (Figure 2).

**Figure 3:**
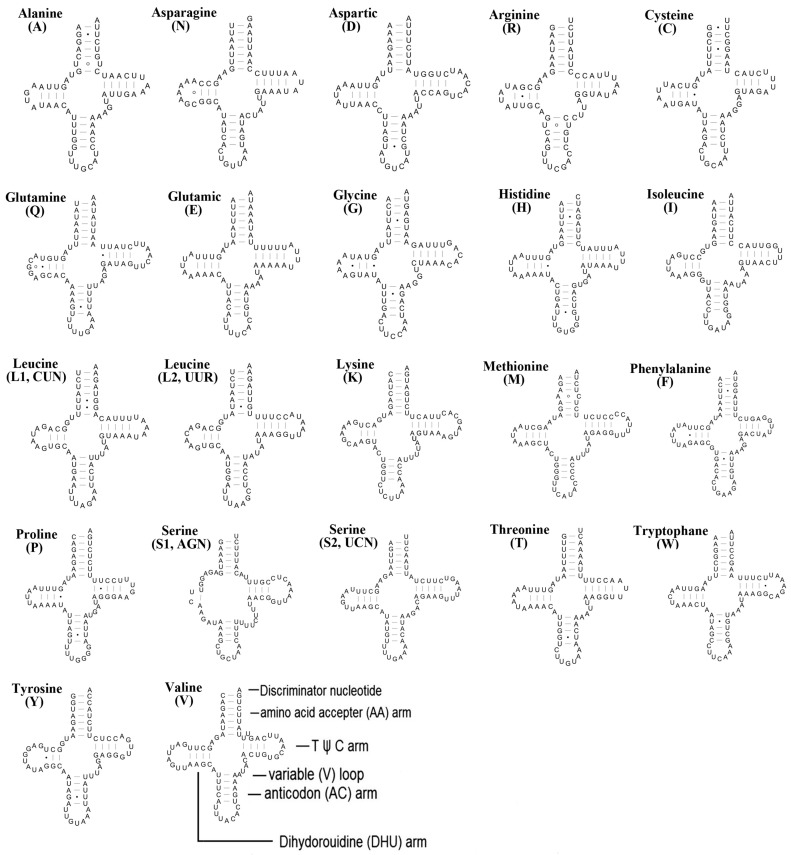
Inferred secondary structures of tRNAs from the *Nemoura nankinensis* mitogenome. The tRNAs are labelled with the abbreviations of their corresponding amino acids. Structural elements in tRNA arms and loops are illustrated as for *trnV*. Dashes (–) indicate Watson–Crick bonds, dots (·) indicate mistaken bonds, and circles (°) indicate loops in arms.

The total length of the 22 tRNA genes was 1404 bp, and individual genes ranged from 63 to 71 bp with an average A+T content of 71.4%. All tRNA genes of *N. nankinensis* were predicted to fold into typical cloverleaf secondary structures (Figure 3). However, in *trnSer* (*AGN*), the dihydrouridine (DHU) arm was reduced to a small loop, which was common in many other metazoan mitogenomes [28]. In addition, 30 mismatched base pairs were identified in the tRNA genes, and these were all G-U pairs. In *N. nankinensis*, the anticodons of the 22 tRNAs were identical with other stoneflies, and the AGG codon was translated as Lys instead of Ser, which indicated the utilization of a variant of the invertebrate mitochondrial genetic code in this mitogenome (Table 1). This phenomenon has been well discussed by Abascal et al., and the shifts between alternative genetic codes were concluded to occur frequently within arthropod main lineages [29,30].

The large ribosomal RNA (*rrnL*) gene of *N. nankinensis* was 1327 bp in length with an A+T content of 74.9%, and the small ribosomal RNA (*rrnS*) gene was 790 bp with an A+T content of 71.3% (Table 1). The two rRNA genes were mapped between *trnLeu* (CUN) and the control region, which was consistent with other stonefly species.

### 2.3. The Control Region

**Figure 4:**
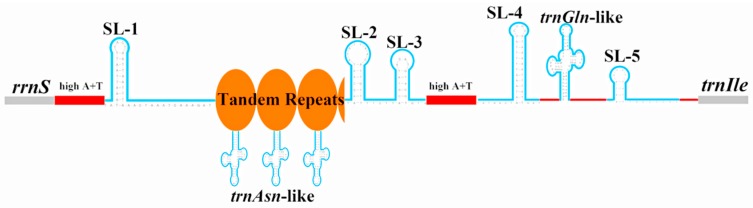
Predicted structural elements in the control region of *Nemoura nankinensis*. Tandem repeat units are indicated by orange ellipses. Stem-loop (SL) and tRNA-like structures are demarcated by blue lines. Other A+T-rich sequences are shown as red lines.

The control region (CR) of the *N. nankinensis* mitogenome is currently the longest known CR (1751 bp) with the highest A+T content (81.8%) among stoneflies, and was located at the conserved position between *rrnS* and *trnIle* (Figure 1, Table 1 and Table 3). Firstly, a large repeat region (15021–16049) was identified, which was 1029 bp and contained 3.1 tandem repeats (Figure 4). Each of the three repeated sequences could be folded into a same *trnAsn*-like structure encoded on the N-strand. These long tandem repeats might explain the large size of the CR in *N. nankinensis*.

**Figure 5:**
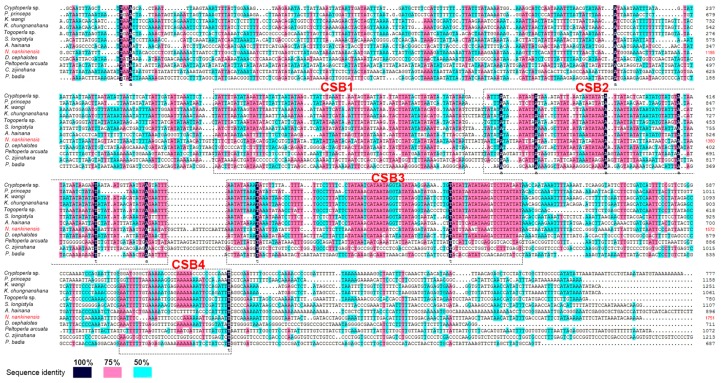
The alignment of conserved structural elements of CRs among stoneflies. Sequence identity among species was indicated by colored boxes. CSB1-4 indicates four conserved sequence blocks in the CRs.

Then five SL structures were predicted in the CR: SL-1 (14,982–15,022), SL-2 (16,064–16,088), SL-3 (16,089–16,110), SL-4 (16,368–16,407) and SL-5 (16,552–16,572). The proposed “G(A)*_n_*T” motif was detected after SL-1 and SL-4, but it was modified as “GTA” after SL-2 and SL-3, and “TGA” after SL-5. These SL structures were considered to be associated with the initiation of mitogenome replication and transcription [31]. Interestingly, a *trnGln*-like structure was found between SL-4 and SL-5, and it was encoded on the majority strand. The presence of tRNA-like structures in the CR has never been reported in other plecopteran mitogenomes, and its underlying mechanisms are unclear. These tRNA-like structures may have signaling functions in the process of transcription [32]. When compared with the available 11 CRs of the other stoneflies, four conserved sequence blocks (CSBs) were identified in *N. nankinensis* (Figure 5). These CBSs ranged in size from 35 to 100 bp, and their sequence identity among species was generally over 50% (Figure 5). However, the function of these CSBs is still unclear.

In the past, the sequenced stonefly mitogenomes were mainly from the superfamily group Systellognatha, only *N. nankinensis* and the three Capniidae species are from the group Euholognatha. The CR of the *Capnia zijinshana* (Plecoptera: Capniidae) mitogenome was completely reported, and was 1513 bp in length, the second longest in stoneflies. Accordingly, we speculate that with more mitogenomes sequenced from Plecoptera, especially from the group Euholognatha, highly varied mitogenome sizes with more novel structural features will be found, and their functions and phylogenetic implications will be clear.

### 2.4. Phylogenetic Analyses

**Figure 6:**
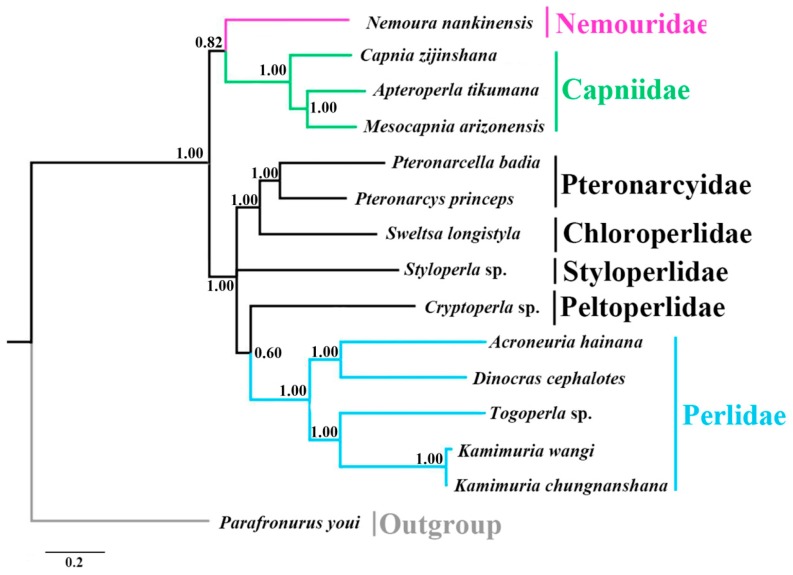
Phylogenetic relationships among stoneflies inferred by Bayesian inference. Numbers at the nodes are posterior probabilities. The family names are listed after the species. The tree was rooted with one outgroup, *Parafronurus youi*.

**Figure 7:**
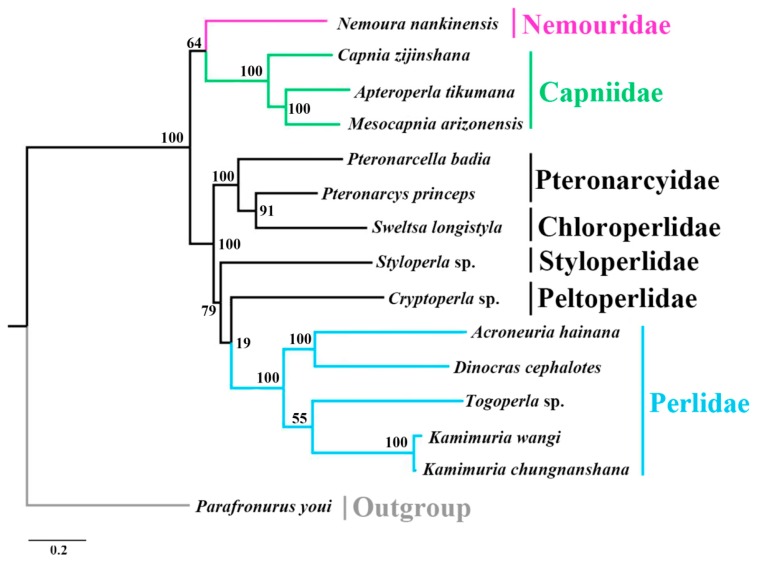
Phylogenetic relationships among stoneflies inferred by maximum likelihood analysis. Numbers at the nodes are bootstrap values. Family names are shown after the species. The tree was rooted with one outgroup, *Parafronurus youi*.

The phylogenetic analyses were performed based on the concatenated nucleotide sequences of 13 PCGs derived from 14 available stonefly mitogenomes, and one Ephemeroptera species was included as the outgroup (Table 3). BI and ML analyses generated similar tree topologies (Figure 6 and Figure 7). In both analyses, *N. nankinensis* was recovered as the sister group of the three species from Capniidae, and the species from other five families were grouped together. This corresponds with the taxonomical knowledge that both Nemouridae and Capniidae are members in the superfamily group Euholognatha, while other five families are from Systellognatha in Plecoptera. In addition to the Capniidae, the clade containing species from Perlidae was well supported on both trees. However, the phylogenetic positions of the four families, Pteronarcyidae, Choloroperlidae, Styloperlidae and Peltoperlidae, were still unclear. These results were generally identical to the recent study made by Chen and Du [12]. The uncertainty and inconsistency of recent mitogenomic phylogenetic studies in Plecoptera may result from the limited mitogenomic data, and more sequencing work is necessary to resolve this problem.

## 3. Materials and Methods

### 3.1. Sample Preparation and DNA Extraction

Specimens of *N. nankinensis* were collected in February 2016 from Zijin Mountain of Jiangsu Province, China. Our research activities were not banned by any organization or individual and did not involve endangered or protected species. Specimens used in this study were identified and preserved in 100% ethanol and stored at −20 °C. Genomic DNA was extracted from adults using the Column mtDNAout kit (Tianda, Beijing, China) and stored at −20 °C until used for PCR.

### 3.2. PCR Amplification and Sequencing

Five pairs of LA-PCR primers were used to amplify segments of the *N. nankinensis* mitogenome (Table A1). Conditions for LA-PCR amplification were as follows: initial denaturation at 93 °C for 2 min, followed by 40 cycles at 92 °C for 10 s; annealing at 54 °C for 30 s; and elongation at 68 °C for 8 min (20 cycles), which increased 20 s/cycle in the final 20 cycles; and final elongation at 68 °C for 10 min. PCR products were separated by 1.0% agarose gel electrophoresis and purified with an Axygen DNA Gel Extraction Kit (Axygen Biotechnology, Hangzhou, China). All PCR fragments were sent to Map Biotech Company (Shanghai, China) for sequencing. Firstly, the LA-PCR fragments were partially sequenced by Shotgun sequencing in combination with the primer walking strategy (Table A1). Then 15 specifically designed primer pairs were used for the remaining gaps including the CR (Table A1).

### 3.3. Mitogenome Assembly, Annotation and Analyses

The software CodonCode Aligner (http://www.codoncode.com/aligner/) was used for sequence assembly. PCGs and rRNA genes were identified by comparison with the previously sequenced stonefly mitogenomes, and the gene boundaries were confirmed with ORF finder (https://www.ncbi.nlm.nih.gov/orffinder/). The mitogenomic map was depicted with CGView Server (http://stothard.afns.ualberta.ca/cgview_server/) [33]. The tRNAs were identified by the online server MITOS combined with ARWEN (http://mbio-serv2.mbioekol.lu.se/ARWEN/) [34,35]. The secondary structure of tRNA genes was also obtained from MITOS. The nucleotide composition was analyzed by MEGA v. 6.0 [36]. Composition skew analysis was performed using the formulas AT-skew = [A–T]/[A+T] and GC-skew = [G–C]/[G+C] [37]. The tandem repeats in the putative control region were analyzed with the Tandem Repeats Finder program (http://tandem.bu.edu/trf/trf.advanced.submit.html) and the stem-loop structures were predicted by Quikfold (http://unafold.rna.albany.edu/?q=DINAMelt/Quickfold) [38]. Sequence data were deposited into GenBank under accession number KY940360.

### 3.4. Phylogenetic Analyses

Phylogenetic analyses were based on nucleotide sequence data of 13 PCGs derived from *N. nankinensis* and 13 other stonefly mitogenomes available from GenBank (Table 3). *Parafronurus youi* (Accession No. EU349015) from the insect order Ephemeroptera was used as the outgroup. The nucleotide sequences of the 13 PCGs were aligned with Clustal X as implemented in MEGA v. 6.0 using default settings before concatenation excluding the stop codon [39]. The length of the alignment was 11,154 nucleotides in the final dataset. The best nucleotide substitution model was determined with MEGA v. 6.0 using the Bayesian Information Criterion (BIC) and the GTR+G+I model was predetermined for analyses. Bayesian inferences (BI) and maximum likelihood (ML) analysis were respectively performed using MrBayes v. 3.1.2 [40] and the RAxML Web-Server (http://embnet.vital-it.ch/raxml-bb/index.php) [41]. The BI analyses were performed under the following conditions: 3 million generations with sampling every 100 generations, four chains (one cold chain and three hot chains) and a burn-in of 25% trees. After 3 million generations, all runs reached in stationarity were examined by Tracer v. 1.5 (effective sample sizes exceed 200) [42]. The confidence values of the BI tree were shown as Bayesian posterior probabilities. One thousand bootstrap replicates were performed with the GTRGAMMA substitution model in ML analyses. Finally, the phylogenetic trees were drawn with the software FigTree v. 1.4.2 [43].

## 4. Conclusions

The mitogenome of *N. nankinensis* was the longest among reported stonefly mitogenomes. The gene arrangement of the *N. nankinensis* mitogenome was highly-conserved and identical with other stoneflies. In the elongated CR, novel features were found, including a large tandem repeat region, five SL structures, four tRNA-like structures and four CSBs. These structural elements may have functions associated with the process of replication and transcription. Phylogenetic analyses supported that Nemouridae was the sister-group of Capniidae, which was consistent with former researches.

## Figures and Tables

**Table 1:** Mitochondrial genome structure of *Nemoura nankinensis*.

Gene	Position (bp)	Size (bp)	Direction	Intergenic Nucleotides (IGN)	Anti- or Start/Stop Codons	AT%
*trnIle (I)*	1–66	66	Forward	0	GAT	66.7
*trnGln (Q)*	64–132	69	Reverse	−3	TTG	78.3
*trnMet (M)*	146–214	69	Forward	13	CAT	62.3
*nad2*	215–1249	1035	Forward	0	ATG/TAA	70.8
*trnTrp (W)*	1259–1327	69	Forward	9	TCA	69.6
*trnCys (C)*	1320–1382	63	Reverse	−8	GCA	68.3
*trnTyr (Y)*	1388–1453	66	Reverse	5	GTA	60.6
*cox1*	1450–2983	1534	Forward	−2	CCG/T-	64.1
*trnLeu2 (UUR)*	2986–3052	67	Forward	0	TAA	68.7
*cox2*	3056–3743	688	Forward	3	ATG/T-	67.2
*trnLys (K)*	3745–3815	71	Forward	1	CTT	64.8
*trnAsp (D)*	3817–3885	69	Forward	1	GTC	73.9
*atp8*	3886–4044	159	Forward	0	ATT/TAA	76.7
*atp6*	4038–4715	678	Forward	−7	ATG/TAA	67.7
*cox3*	4715–5503	789	Forward	−1	ATG/TAA	64.9
*trnGly (G)*	5504–5569	66	Forward	0	TCC	72.7
*nad3*	5570–5923	354	Forward	0	ATT/TAG	72.0
*trnAla (A)*	5922–5985	64	Forward	−2	TGC	70.3
*trnArg (R)*	5986–6049	64	Forward	0	TCG	64.1
*trnAsn (N)*	6052–6117	66	Forward	2	GTT	72.7
*trnSer1 (AGN)*	6118–6184	67	Forward	0	GCT	67.2
*trnGlu (E)*	6185–6250	66	Forward	0	TTC	90.9
*trnPhe (F)*	6249–6315	67	Reverse	−2	GAA	67.2
*nad5*	6316–8050	1735	Reverse	0	ATG/T-	70.7
*trnHis (H)*	8051–8117	67	Reverse	0	GTG	79.1
*nad4*	8119–9459	1341	Reverse	1	ATG/TAA	71.5
*nad4l*	9453–9749	297	Reverse	−7	ATG/TAA	74.7
*trnThr (T)*	9752–9817	66	Forward	2	TGT	80.3
*trnPro (P)*	9817–9882	66	Reverse	−1	TGG	71.2
*nad6*	9884–10408	525	Forward	1	ATT/TAA	73.3
*Cytb*	10408–11544	1137	Forward	−1	ATG/TAG	66.8
*trnSer2 (UCN)*	11543–11612	70	Forward	−2	TGA	74.3
*nad1*	11644–12594	951	Reverse	31	TTG/TAA	69.4
*trnLeu1 (CUN)*	12596–12661	66	Reverse	1	TAG	75.8
*rrnL*	12664–13990	1327	Reverse	2		74.9
*trnVal (V)*	13991–14061	71	Reverse	0	TAC	69.0
*rrnS*	14062–14851	790	Reverse	0		71.3
Control region	14852–16602	1751		0		81.8

**Table 2:** Nucleotide composition of the *Nemoura nankinensis* mitogenome.

Regions	Nucleotides Proportions (%)						AT Skew	GC Skew
	A	T	G	C	A+T	G+C		
Whole genome	37.2	34.0	11.8	17.0	71.2	28.8	0.04	−0.18
Protein coding genes	35.7	33.3	12.9	18.1	69.0	31.0	0.03	−0.17
1st codon position	40.5	29.3	14.4	15.8	69.8	30.2	0.16	−0.05
2nd codon position	31.5	32.2	15.3	21.0	63.7	36.3	−0.01	−0.16
3rd codon position	35.1	38.3	9.1	17.5	73.4	26.6	−0.04	−0.32
Protein coding genes-J	29.7	38.0	14.1	18.2	67.7	32.3	−0.12	−0.13
1st codon position	30.5	35.0	17.6	16.9	65.5	34.5	−0.07	0.02
2nd codon position	26.4	40.5	12.5	20.6	66.9	33.1	−0.21	−0.24
3rd codon position	32.2	38.5	12.2	17.1	70.7	29.3	−0.09	−0.17
Protein coding genes-N	45.3	25.6	11.1	18.0	70.9	29.1	0.28	−0.24
1st codon position	46.9	26.2	9.7	17.2	73.1	26.9	0.28	−0.28
2nd codon position	45.7	25.5	11.0	17.8	71.2	28.8	0.28	−0.24
3rd codon position	43.2	25.2	12.6	19.0	68.4	31.6	0.26	−0.20
tRNA genes	36.8	34.6	12.8	15.8	71.4	28.6	0.03	−0.10
tRNA genes-J	36.4	34.9	14.7	14.0	71.3	28.7	0.02	0.02
tRNA genes-N	37.7	33.8	9.1	19.4	71.5	28.5	0.05	−0.36
rRNA genes	40.3	33.2	9.6	16.9	73.5	26.5	0.10	−0.28
Control region	43.2	38.7	6.4	11.7	81.9	18.1	0.05	−0.29

**Table 3:** Comparison of mitogenome size and control region size with A+T content among stoneflies.

Species	Mitogenome Size (bp)	Control Region Size (bp)	A+T Content of Control Region (%)	Accession Number
*N. nankinensis*	16,602	1751	81.8	KY940360
*C. zijinshana*	16,310	1513	62.0	KX094942
*M. arizonensis*	14,921	N/A	N/A	KP642637
*A. tikumana*	15,564	N/A	N/A	KR604721
*Styloperla* sp.	15,416	N/A	N/A	KR088971
*S. spinicercia*	16,129	1259	77.3	KX845569
*Cryptoperla* sp.	15,633	777	80.2	KC952026
*S. longistyla*	16,151	1107	80.1	KM216826
*P. princeps*	16,004	1158	81.3	AY687866
*P. badia*	15,586	687	68.9	KU182360
*D. cephalotes*	15,666	711	74.5	KF484757
*A. hainana*	15,804	899	73.3	KM199685
*Togoperla* sp.	15,723	780	78.0	KM409708
*K. wangi*	16,179	1251	78.2	KC894944
*K. chungnanshana*	15,943	1062	79.1	KT186102
*Peltoperla arcuata*	N/A	1072	N/A	AY142073

N/A: data not available.

## Abbreviations

IGN	Intergenic Nucleotide
PCG	Protein-Coding Gene
tRNA	Transfer RNA
rRNA	Ribosomal RNA
CR	Control Region
SL	Stem-Loop
CSB	Conserved Sequence Block
BI	Bayesian inferences
ML	Maximum likelihood

## Appendix A

**Table A1:** Sequencing strategy and primers used in this study.

Regions	Strategy	LA-PCR Primers (5′–3′)	Specific Primers (5′–3′)
*cox1*–*cytb*	Shotgun	COI-1F: GAAAATGGGGCTGGGACCG Cytb-1R: TTCGGGTTGAATATGAACAGGG	YP01200761-1F: AGAAGACCGACGAACGCC YP01200765-1F: CAGCAAAAGGCCCCCACG YP01200769-1F: CGGTTTACAGCAGCACTTCA YP01200786-1F: CCCTTTTAGACACCACCCTT YP01200797-1F: TACAGCTGGTAAAAATAGTTCAAA YP01200760-1R: TTCACACAAGCTGCACGAT YP01200772-1R: TGTTGGGAAGAGGAATCTAAGA YP01200773-1R: AACTGCCTGTGGGTATCGT YP01200783-1R: GCCGAAACCCCCCTCCTG YP01200786-2F: TTGCCATTCCAAACCCAT YP01200783-1F: TGCGTTTCGGCAAGGTAT YP01200797-1R: GGTTGATGCTACCCCTGG NJCJ-COI-3F: GATTTATGCCATCACGATACTCA NJCJ-COI-5F: ATGCTTTATCCCATTTTTCG NJCJ-Cytb-4R: TTTGGGTTGTGATGTTATTTCTT NJCJ-COI-5R: TGTTGGGGAAAGTCTTCTATGGA NJCJ-COI-6F: AAAAGCGGCATATCACTGTT NJCJ-Cytb-5R: TTGATTGCTTACTCTTCGGTTG NJCJ-COI-7F: ACCCCAATCAGCCTCCTAA NJCJ-Cytb-6R: GTTGTTTTTACATTGATTTCCTTT
	Primer walking	COI-2F: GGTTGATGCTACCCCTGGACG COI-4F: TTGATTTCACCCAATATAGAACTAA Cytb-3R: GGGGATGTGTTATTTGGGTG Cytb-2R: GAAAGCTAAGTCAATATGGGCA Cytb-4F: GCCTAATGCCCCCTCACA COI-5R: TGTTGGGGAAAGTCTTCTATGGA	
*cytb*–*cox1*	Shotgun	Cytb-1F: TTCATCTCCTATTCCTTCACCAAA COI-1R: GTGATTGCTACGGCTCAAACGAA	YP01190217-1F: TGGTCGGGGTTGCTTTCT YP01190203-1R: ATAATTTCCCGATTTAAAGAGAG YP01190233-1R: TTCGTAAGCTACACCTTGACC YP01190202-1F: GGCACTTCTGCTCTTCCCG YP01190227-1F: AAAGTCTTCGATCTGTCAGTTGT YP01190213-2R: CGTAAGAAAAACAATGTTAATATA YP01190213-3R: ATATATTATATCTATATGGATATTATGTTT NJCJ-control-1F: TATGGTTCTATATGCAATTTCCTCCC YP01190213-1R: TAGAGAGAAGAGGGGTAAAAC
